# Supplementary material for: All-at-once RNA folding with 3D motif prediction framed by evolutionary information
Source: Nat Methods. 2025 Oct 3;22(10):2094–106. doi: 10.1038/s41592-025-02833-w (PMC12510878; doi:10.1038/s41592-025-02833-w)
Supplement: Supplementary file 3 — Source code to run CaCoFold-R3D. Data and results. [file 41592_2025_2833_MOESM3_ESM.gz › supplemental_material/results/CaCoFold-R3D/CaCoFold-R3D_Rfam/RF02119_FTX_1.R2R.sto.pdf]

RF02119_FTX_1

5′ – A ● U G A G A U R Y C U G U U Y C U U U G U C C A ● A U C ● C ● U U U U R A G A U A U C A A U G G A A R R A U U A A U U C C U C Y U A A U C C
